# Quantification of cervical spinal stenosis by automated 3D MRI segmentation of spinal cord and cerebrospinal fluid space

**DOI:** 10.1038/s41393-024-00993-8

**Published:** 2024-04-16

**Authors:** Marc Hohenhaus, Jan-Helge Klingler, Christoph Scholz, Ralf Watzlawick, Ulrich Hubbe, Jürgen Beck, Marco Reisert, Urs Würtemberger, Nico Kremers, Katharina Wolf

**Affiliations:** 1 Department of Neurosurgery, Medical Center – University of Freiburg, Faculty of Medicine, University of Freiburg, Breisacher Straße 64, 79106 Freiburg, Germany; 2 Department of Radiology, Medical Physics, Medical Center – University of Freiburg, Faculty of Medicine, University of Freiburg, Killianstraße 5a, 79106 Freiburg, Germany; 3 Department of Neuroradiology, Medical Center – University of Freiburg, Faculty of Medicine, University of Freiburg, Breisacher Straße 64, 79106 Freiburg, Germany; 4 Department of Neurology, Medical Center – University of Freiburg, Faculty of Medicine, University of Freiburg, Breisacher Straße 64, 79106 Freiburg, Germany

**Keywords:** Spinal cord diseases, Spinal cord, Magnetic resonance imaging

## Abstract

**Design:**

Prospective diagnostic study.

**Objectives:**

Anatomical evaluation and graduation of the severity of spinal stenosis is essential in degenerative cervical spine disease. In clinical practice, this is subjectively categorized on cervical MRI lacking an objective and reliable classification. We implemented a fully-automated quantification of spinal canal compromise through 3D T2-weighted MRI segmentation.

**Setting:**

Medical Center - University of Freiburg, Germany.

**Methods:**

Evaluation of 202 participants receiving 3D T2-weighted MRI of the cervical spine. Segments C2/3 to C6/7 were analyzed for spinal cord and cerebrospinal fluid space volume through a fully-automated segmentation based on a trained deep convolutional neural network. Spinal canal narrowing was characterized by relative values, across sever segments as adapted Maximal Canal Compromise (aMCC), and within the index segment as adapted Spinal Cord Occupation Ratio (aSCOR). Additionally, all segments were subjectively categorized by three observers as “no”, “relative” or “absolute” stenosis. Computed scores were applied on the subjective categorization.

**Results:**

798 (79.0%) segments were subjectively categorized as “no” stenosis, 85 (8.4%) as “relative” stenosis, and 127 (12.6%) as “absolute” stenosis. The calculated scores revealed significant differences between each category (*p* ≤ 0.001). Youden’s Index analysis of ROC curves revealed optimal cut-offs to distinguish between “no” and “relative” stenosis for aMCC = 1.18 and aSCOR = 36.9%, and between “relative” and “absolute” stenosis for aMCC = 1.54 and aSCOR = 49.3%.

**Conclusion:**

The presented fully-automated segmentation algorithm provides high diagnostic accuracy and objective classification of cervical spinal stenosis. The calculated cut-offs can be used for convenient radiological quantification of the severity of spinal canal compromise in clinical routine.

## Introduction

The evaluation of individual anatomy is essential for treatment of patients with degenerative cervical spine disease starting with degenerative cervical spondylosis and ending up in evident degenerative cervical myelopathy (DCM).

Several pathophysiological factors are important for the assessment of degenerative cervical spine disease and can be visualized using different Magnetic Resonance Imaging (MRI) techniques [1–3]. Essential is the anatomical configuration of the spinal canal and its narrowing, which is commonly evaluated on conventional T2-weighted sequences. As a result of the spinal canal compromise, different stages of spinal cord affection appear and can be visualized using advanced MRI sequences [2]. Severe spinal cord damage can be already visualized on conventional T2-weighted sequences as hyperintensities and T1-weighted sequences as hypointensities, consequently leading to spinal cord atrophy [4]. The correlation of imaging alterations and symptoms of affected patients is heterogeneous and it is crucial to evolve the links between imaging and its clinical and prognostic value to implement the optimal treatment [5, 6].

The severity of a spinal stenosis is traditionally classified subjectively in poorly delineable categories by radiologists in clinical routine, whereas a more precise graduation could be useful for dedicated treatment planning. Additionally, an objective and reproducible classification is necessary to standardize research evaluations beside patients’ care.

**Table 1:** Inter-observer statistics for subjective spinal stenosis categorization by all three raters (Intra-class Correlation Coefficient (ICC), two-way model, absolute agreement, single-measurements, mixed effects model).

Cervical level	C2/3	C3/4	C4/5	C5/6	C6/7
Absolute agreement of all three raters	199/202 (98.5%)	185/202 (91.6%)	179/202 (88.6%)	166/202 (82.2%)	175/202 (86.6%)
ICC	0.869	0.921	0.932	0.916	0.885
p-value	<0.001	<0.001	<0.001	<0.001	<0.001

**Table 2:** Subjective consensus spinal stenosis categorization separated for the different cervical levels (*n* = 202 per level).

Cervical Level	C2/3	C3/4	C4/5	C5/6	C6/7
No stenosis	200 (99.0%)	158 (78.2%)	156 (77.2%)	121 (59.9%)	163 (80.7%)
Relative stenosis	0	25 (12.4%)	12 (5.9%)	31 (15.3%)	17 (8.4%)
Absolute stenosis	2 (1%)	19 (9.4%)	34 (16.8%)	50 (24.8%)	22 (10.9%)

The aim of this study was to standardize and objectively quantify the spinal canal compromise by implementing a fully-automated classification based on 3D segmentations of the spinal cord and cerebrospinal fluid (CSF) space on routinely-applicable, high-resolution T2-weighted MRI.

## Methods

### Study outline

This pooled data analysis was based on 114 patients affected from degenerative cervical spine disease and 88 asymptomatic volunteers that had been enrolled in two independent prospective trials, receiving an identical T2-weighted MRI sequence of the cervical spine. Included patients had to provide symptoms of degenerative cervical spine disease and radiographic disc degeneration with contact to the spinal cord or spinal stenosis with cord compression [7]. Myelopathic symptoms were not necessary for inclusion. Patients with contraindication for MRI, previous surgery of the cervical spine as well as non-degenerative alterations (tumor, inflammation, infection, trauma) or relevant multilevel spinal cord compression were excluded [7]. Healthy participants had to be asymptomatic without any neurological disease or severe comorbidity. The studies were approved by the institutional ethics committee (references 261/17 and 338/17) and registered at the National Clinical Trials Registry (DRKS00012962, DRKS00017351). A signed informed written consent was obtained from each participant prior to inclusion in both trials. MRI scans between 04/2018 and 01/2022 were included for evaluation.

### MRI specifications

All MRI scans were performed with a 3 T MRI scanner (MAGNETOM Prisma, Siemens Healthcare, Erlangen, Germany) with a 64-channel head-neck coil for the cervical spine with a clinical standardized T2-weighted 3D sequence (T2 SPACE, voxel size 0.6 mm × 0.6 mm × 1.0 mm, TR 1500 ms, TE 134 ms, Flip angle 105°, GRAPPA PAT: 3, acquisition time 3:53 min).

### Automated segmentation process

**Fig. 1:**
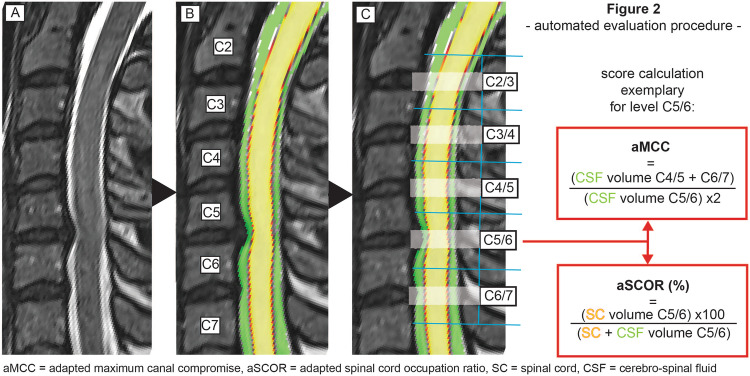
Overview of the fully-automated evaluation procedure of an exemplary patient with cervical stenosis in C5/6. **A** High-resolution 3D T2-weighted images. **B** Segmentation of spinal cord (yellow) and CSF space (green) with determination of the vertebral bodies from C2 to C7. **C** Calculation of CSF and spinal cord volumes at the middle third of each evaluated segment (white shaded rectangle). Right: Formula for aMCC (CSF space proportion of the index to both surrounding segments) and aSCOR (proportion of spinal cord and CSF space at the index segment) as objective parameters.

All MRI datasets were segmented fully-automated for the cross-sectional area (CSA, mm²) of CSF space and spinal cord per slice from C2 to C7 through a specialized in-house software pipeline (NORA framework, www.nora-imaging.org). The segmentation process was conducted by a trained deep convolutional neural network based on an U-net type architecture [8]. The applied algorithm is provided online (https://bitbucket.org/reisert/patchwork/wiki/Home). The convolutional neural network was trained on 125 cases and validated using five separate cases, all out of the study dataset randomly. Dice coefficients for the spinal cord of 0.94 (±0.01) and for CSF space of 0.90 (±0.03) were reached, which was comparable to the recent literature [9, 10]. To quantify and compare spinal cord and CSF space volumes in a common reference space, the cervical vertebral bodies C2 to C7 were annotated and trained by another patchwork convolutional neural network to allow automated detection. Using the localization of the vertebral bodies, the images were straightened along the cord axis and spinal cord and CSF space CSA were computed. Therefore, the specified image resolution is affected of a slightly anatomic distortion of the primary radiological 1 mm slice thickness. The segmentation was integrated in a routinely usable visualization (Fig. 1). All segmentations were checked concerning apparent errors independently by two examiners, whereas no measurement had to be excluded due to misssegmentation.

### Calculation of adapted Maximal Canal Compromise (aMCC) and adapted Spinal Cord Occupation Ratio (aSCOR)

Value curves for CSF space CSA and spinal cord CSA were generated from C2 to C7 slice by slice. For evaluation of the occurrence of a spinal stenosis, two already established parameters reflecting spinal canal compromise were automatically calculated: the adapted Maximal Canal Compromise (aMCC) and the adapted Spinal Cord Occupation Ratio (aSCOR). The aMCC is defined as sum of the spinal canal CSA one segment above and below divided through the doubled spinal canal CSA at the addressed level [1, 11]. The higher the aMCC, the higher the degree of the spinal stenosis. The spinal canal was defined as sum of CSF and spinal cord CSA. The aSCOR relates the CSA of CSF space and spinal cord at the addressed level. It is calculated as the percentage of spinal cord CSA to the total spinal canal [12, 13]. The higher the aSCOR, the higher the spinal stenosis, similar to the aMCC.

For the calculation of both scores, we focused on the “middle third” between the centers of two vertebral bodies, like depicted in Fig. 1C, reflecting the level of the intervertebral disc as commonly pathologically affected area. An overview of the complete evaluation procedure including score definition is illustrated in Fig. 1. The post-processing with segmentation and calculation of both scores after image acquisition takes about 3 min.

### Subjective categorization of spinal stenosis

**Fig. 2:**
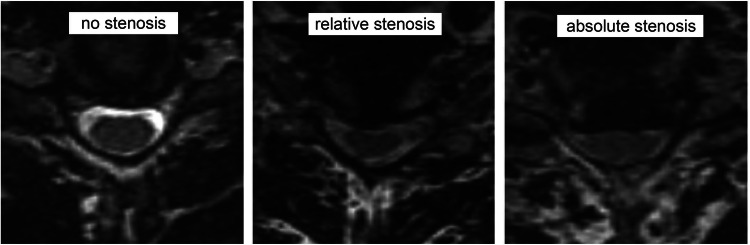
Subjective categorization of cervical spinal stenosis. Exemplary transverse T2-weighted images at level C5/6 for all three subjective stenosis categories: “no stenosis” = no degenerative elements contacting the spinal cord; “relative stenosis” = focal narrowing of the CSF space with contact to the spinal cord or circumferential narrowing with residual CSF signaling; “absolute stenosis” = absent CSF space with or without spinal cord volume reduction.

To achieve ground truth, all MRI scans were subjectively evaluated concerning presence and severity of a spinal stenosis for each cervical segment from C2 to C7 through three independent observers, each providing more than ten years experience in spinal imaging. This resulted in five ratings (C2/3, C3/4, C4/5, C5/6, C6/7) per MRI scan and observer. For the subjective grading, we applied common clinically used categories adapted to Kang et al. [14] as follows: “no” stenosis (no degenerative elements contacting the spinal cord), “relative” stenosis (focal narrowing of the CSF space with contact to the spinal cord or circumferential narrowing with residual CSF signaling) and “absolute” stenosis (absent CSF space with or without spinal cord volume reduction). Exemplary cases are shown in Fig. 2. Solely the acquired T2 SPACE sequence was used for subjective grading and the observers were blinded to clinical data of the evaluated measurements.

The inter-rater reliability of all three observers was evaluated through Intra-class Correlation Coefficient (ICC) statistics and qualified according to Koo et al. [15]: ICC < 0.50 = poor, ICC 0.50–0.75 = moderate, ICC 0.75–0.90 = good, ICC > 0.90 = excellent.

For further evaluations, a consensus in case of disagreement of the observers was forced through the assumption of the categorization of the two matching observers, fading-out the third aberrant rating.

### Statistical analysis

The statistical evaluation was performed by IBM SPSS Statistics 27. Normal distribution for each variable was assessed by Shapiro–Wilk-Test. Group comparison was done by Mann–Whitney-U-test for independent samples for nominal variables and Kruskal–Wallis-test for ordinal variables. The optimal cut-off values for aMCC and aSCOR to separate for “no”, “relative” and “absolute” stenosis were approximated by calculation of the Youden’s Index of Receiver Operating Characteristic (ROC) analysis. The two-way model with absolute agreement for single-measurements and multiple raters in an ordinal scale was applied for ICC statistics [16].

## Results

### Baseline characteristics

A total of 202 datasets were available, consisting of 114 (56.4%) patients with symptoms due to cervical degenerative spine disease and 88 (43.6%) asymptomatic volunteers. Median age of all included participants at the date of MRI was 55.0 [IQR 41.8–63.9] years and 94 (46.5%) were female. Age distribution showed significantly younger healthy volunteers (median 50.5 [IQR 31.0–62.0] years) compared to the included symptomatic patients (median 58.0 [IQR 48.0–66.3] years, *p* = 0.001). All symptomatic patients and even 20 (22.7%) of the 88 asymptomatic volunteers showed a spinal stenosis at any cervical level.

For further evaluation, each cervical segment was evaluated concerning the extent of the spinal canal compromise regardless of the clinical status.

### Subjective categorization of spinal stenosis

A total of 1010 cervical segments were subjectively categorized by each of the observers. Identical ratings from all three observers occurred in 89.5% (904/1010 cervical segments). Table 1 shows the number of absolute agreements and the associated ICCs separated for the different cervical levels, indicating good to excellent results (0.869–0.932, *p* < 0.001).

After achieving consensus from the three independent ratings, 798 (79.0%) of all evaluated 1010 cervical segments showed “no” stenosis, whereas 85 (8.4%) showed a “relative” and 127 (12.6%) an “absolute” spinal stenosis. The distribution for the different cervical levels is shown in Table 2, presenting a predominantly pathologically affected level C5/6 (40.1%), followed by C3/4 (21.8%) and C4/5 (22.8%).

### Spinal stenosis classification by adapted Maximal Canal Compromise (aMCC)

**Fig. 3:**
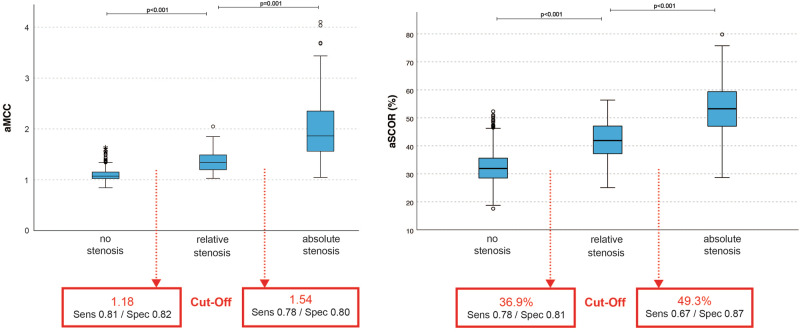
Boxplots for aMCC and aSCOR values. Comparison using Kruskal–Wallis test for independent samples revealed significant differences between all evaluated groups (*p* ≤ 0.05). The cut-offs between the groups (rectangle) were determined by ROC analysis and calculation of Youden’s Index.

**Fig. 4:**
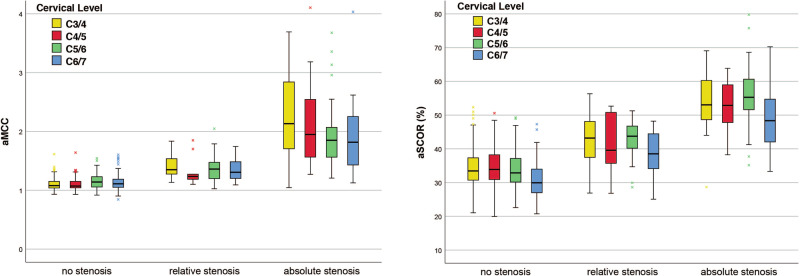
Boxplots for aMCC and aSCOR values separated for the different cervical levels: yellow = C3/4, red = C4/5, green = C5/6, blue = C6/7. Level C2/3 is not depicted for clarity reasons, because of only two patients with a pathological affected segment. Absolute values and significance levels comparing the three subjective categories are added as Supplement 2.

Dividing all cervical segments respective their subjective stenosis categorization, there were significantly increasing median aMCC values for progressive cervical stenosis (“no” stenosis 1.07 [IQR 1.02–1.15] vs. “relative” stenosis 1.34 [IQR 1.20–1.49] vs. “absolute” stenosis 1.86 [IQR 1.56–2.37], *p* < 0.001 and *p* = 0.001, Fig. 3). Calculation of Youden’s Index revealed an optimal cut-off to separate “no” from “relative” stenosis for an aMCC of 1.18, reaching a sensitivity of 81% and specificity of 82%. For “absolute” stenosis the cut-off was expectably higher at 1.54 (sensitivity 78%, specificity 80%, Fig. 3). ROC curves are added as Supplement 1. We additionally separated the median aMCC values for the single cervical levels, revealing persistent significant differences for the differentiation of the stenosis categorization (all *p* < 0.001), except for distinguishing “relative” from “absolute” stenosis in C3/4 (1.35 [IQR 1.27–1.55] vs. 2.13 [1.70–2.97], *p* = 0.286) and C6/7 (1.31 [1.20–1.49] vs. 1.82 [1.40–2.26], *p* = 0.173). Boxplots are shown in Fig. 4 and absolute values are added as Supplement 2.

### Spinal stenosis classification by adapted Spinal Cord Occupation Ratio (aSCOR)

Similar to the aMCC, the evaluation revealed significantly increasing median aSCOR values for progressive cervical stenosis (“no” stenosis 31.90% [IQR 28.47–35.60] vs. “relative” stenosis 41.89% [IQR 37.16–47.15] vs. “absolute” stenosis 53.26% [IQR 46.95–59.64], both *p* < 0.001, Fig. 3). Separating for the different cervical levels showed corresponding significant results, except for distinguishing “relative” from “absolute” stenosis in C6/7, barely missing the level of statistical significance (38.52% [34.03–44.58] vs. 48.34% [41.88–54.74], *p* = 0.053, Fig. 4, Supplement 2). Optimal cut-off values were determined by Youden’s Index for an aSCOR of 36.9% for “no” to “relative” stenosis (sensitivity 78%, specificity 81%) and 49.3% for “relative” to “absolute” stenosis (sensitivity 67%, specificity 87%, Fig. 3). The ROC curves for aSCOR are attached as Supplement 1 as well.

## Discussion

To gain dedicated anatomical classification in patients with degenerative cervical spine disease, we implemented a fast and routinely applicable, fully-automated segmentation of CSF space and spinal cord to determine the severity of spinal canal compromise based on a high-resolution MRI sequence. We established cut-off values for aMCC and aSCOR to separate patients without stenosis and relative to absolute spinal stenosis. This quantification algorithm is a milestone in objective and reproducible radiological assessment in patients with suspected DCM and for the first time implemented into clinical routine diagnostics.

To gain the application into a clinical routine setting, the procedure has to be quick, reliable and convenient.

The acquisition time for our sequence of 3:53 min in combination with a post-processing of 3 min reflects an appropriate length as required for daily clinical practice. Spinal cord and canal segmentation has been described previously, whereas automated segmentation procedures are still under investigation. De Leener et al. [10] reported a reliable segmentation process with Dice coefficients of 0.91 for 18 subjects. However, they stated a notably longer acquisition time of 22 min and focused on the process technology without linking to a clinical routine application. The CSF segmentation procedure and volume calculation for 13 patients with spontaneous intracranial hypotension and 12 healthy volunteers by Fu et al. showed also sufficient accuracies (Jaccard coefficients >0.9) using an artificial intelligence model with a similar segmentation process through U-net architectures [17, 18]. They reported no information concerning the duration of the image acquisition or post-processing. Segmentations using convolutional neural networks solely for the spinal cord are described for intramedullary tumors, multiple sclerosis or other inflammatory diseases, whereas the application in DCM is still rare [19–21].

For 3D segmentation, a slice thickness of at maximum 1 mm seems to be recommendable, with the drawback of a worse but adequate signal-to-noise-ratio and image contrast [22]. The diagnostic quality for intramedullary signal alterations could be limited, which has to be addressed through further evaluations. Two independent examiners reviewed all segmentations concerning errors, without detecting unacceptable results that had to be excluded from further analysis. Still, minor segmentation errors, like aberrations due to strong flow artifacts through CSF pulsation resulting in T2 signal reduction at the CSF space as well as two patients showing a prominent central canal, were seen. In addition, large T2 hyperintensities of the spinal cord due to myelopathy could complicate the delineation.

The traditionally applied, subjective categorization of cervical stenosis is lacking standardized comparability. Several subjective scores have been reported, but none was implemented by default. The most frequently applied graduation introduced by Kang et al. [14] includes four grades: absence of stenosis, CSF space reduction of more than 50%, additional spinal cord deformity, and at least an associated spinal cord T2 signal change. The inter-observer heterogeneity of subjective classifications is a well-known problem. For these categories, an inter-observer agreement was stated with about 60% and ICC values of 0.716–0.737, depending on the cervical level [14, 23]. For other classifications similar results were reported [23, 24]. We applied a three-step classification system adapted to Kang et al., exclusively facing the anatomic canal configuration without regard of intramedullary signal changes. The inter-observer evaluation of our subjective categorization showed good to excellent results (ICC 0.869–0.932), slightly superior to the literature [14, 23]. But there was still incongruence in 10.5% of the cases. So, the purpose of an automated quantification of the spinal canal narrowing is to eliminate subjectivity and therefore observer dependency. The usage of a more subtle, subjective classification for spinal stenosis might be interesting as well, especially to evaluate the 3D segmentation with aMCC and aSCOR cut-offs, but unfortunately there is no such a validated score within the recent literature.

As objective parameters, we used already established parameters adapted to the assessment of cross-sectional areas [1, 11–13]. To take intra- and inter-individual variations of the spinal cord and canal dimension into account, both scores are calculated within the same measurement and not dealing with absolute or normalized external values. Within a review of Frostell et al. [25] the spinal cord extension was characterized with an intra-individual variation of about 20% in transverse and 10% in anteroposterior diameter through the different cervical levels. The inter-individual heterogeneity is quite higher up to 25–30% [25, 26]. Cadotte et al. [27] evaluated the segmental intramedullary configuration and distance to the exiting spinal nerve rootlets through a high-resolution MRI, whereas no information concerning spinal cord and canal dimension were provided. To our knowledge, there are currently no more precise 3D data concerning the morphology of the cervical spinal cord and CSF space.

A limitation for the aMCC is, that this parameter deals with the segments surrounding the index level, leading to bias if applied in multilevel spinal stenosis. In our cohort, we evaluated predominantly monolevel affected patients. The robustness of this segmentation procedure and the applied parameters have to be further investigated for severe multilevel degeneration, because many patients suffer from multisegmental affection. Additionally, both applied scores were calculated for the area surrounding the intervertebral disc, as common stenotic region (Fig. 1C). Alternatively, taking the whole segment for calculation might include larger, mostly unaffected areas directly behind the vertebral body [28]. At least, the aMCC and aSCOR are arbitrarily chosen variables to classify spinal stenosis, whereas other parameter patterns are imaginable triggered by this high-resolution imaging.

The stated cut-off values for aMCC and aSCOR reached acceptable diagnostic accuracies (Fig. 3, Supplement 1), but they have to be reassessed through further datasets and additionally correlated to clinical symptoms of affected patients. One of the limitations of our study is, that we are dealing with solely radiological data without correlation to clinical affection of the measured participants. This is part of ongoing investigations. Additionally, the intramedullary T2 signal intensity has to be included into the radiological classification of cervical stenosis, which was already subject of a prior evaluation of our research group [29]. We are aware that we only included measurements from a single institution using the same imaging protocol. In summary, to bring the provided classification algorithm into a broad usage, it has to be reevaluated through larger patient cohorts and validated through external and also ideally more subjective expert observers.

For future prospects, the presented segmentation allows for 3D shape reconstruction of CSF space and spinal cord in its entirety. Even the applied three-step classification is a first approach to classify the anatomy of DCM patients. But already small osteophytes with distinct spinal cord impression can cause neuronal dysfunction without circumferential compression (Supplement 3). DCM is known as a chronically progressive disease and the course of the patients is not well defined and predictable. In case of symptom deterioration, the detection of distinct progressive spinal cord compression could be of importance, which is sometimes hard to distinguish subjectively on the associated images. So, establishing a subtle, objective classification for every cervical level could add relevant information. Additionally, finding radiological alterations in subclinical affected patients with specific prognostic constellations, would be desirable. Basically, a more detailed description of pathological alterations seems to be necessary for optimal treatment planning. And, as already mentioned above, the evaluation of the shape of the entire spinal cord and canal has to be addressed in clinical routine, because many DCM patients suffer from multisegmental spinal canal stenosis in different dimensions, which was not adequately represented by the patient cohort of this work.

At least, we have to integrate information beside the anatomical canal compromise. Even diffusion parameters from diffusion-weighted sequences, CSF and spinal cord motion depicted by phase-contrast imaging or metabolite configurations using MR spectroscopy could be integrated into a holistic imaging evaluation of DCM patients [2, 7, 30]. Afterwards, such a multimodal MRI work-up has to be correlated to the patients’ symptoms and electrophysiology to optimize clinical treatment decisions and outcomes. In order to achieve this goal in the foreseeable future, multicenter and probably international cooperations are indispensable like in other “big data” projects. The anatomical classification of the spinal canal compromise is a crucial requirement for such a multimodal approach, whereas our evaluation adds the first fast and reliable, fully-automated quantification algorithm for spinal stenoses in a routinely clinical fashion.

## Conclusion

The presented fast and fully-automated 3D MRI segmentation algorithm provides high diagnostic accuracy for an objective classification of cervical spinal stenosis in this monocentric approach. The calculated cut-offs can be used to quantify radiological severity of spinal stenosis in clinical routine, receiving a reproducible and objective grading. An advanced classification system for a more detailed description of spinal stenosis is already under investigation based on these 3D anatomical data for an improved understanding of local pathophysiology and treatment decision-making in affected patients. Nevertheless, the evaluation pipeline has to be validated for external images as well as other MRI protocols before getting into clinical routine and extended for the application in multisegmental affected patients.

### Supplementary information

Supplementary Legend

Supplement 1

Supplement 2

Supplement 3

## Data Availability

The datasets generated and analyzed during the current study are available from the corresponding author on reasonable request.
