# Supplementary material for: Quantification of cervical spinal stenosis by automated 3D MRI segmentation of spinal cord and cerebrospinal fluid space
Source: Spinal Cord. 2024 Apr 16;62(7):371–7. doi: 10.1038/s41393-024-00993-8 (PMC11230899; doi:10.1038/s41393-024-00993-8)
Supplement: Supplementary file 1 — Supplementary Legend [file 41393_2024_993_MOESM1_ESM.docx]

**Supplement 1:** ROC curves for aMCC and aSCOR for differentiation of the subjective categories of spinal canal compromise.

**Supplement 2**: Absolute values and significance levels between the three subjective categories separated for all evaluated cervical levels for adapted Maximal Canal Compromise (aMCC) and adapted Spinal Cord Occupation Ratio (aSCOR). p<0.05 is stated as statistically significant and marked as bold type.

**Supplement 3:** Sagittal and transverse T2-weighted images of three exemplary patients with subjective "relative stenosis" categorization, but different compression pattern on the spinal cord: Patient (A) shows a slightly disc protrusion with contact but without deforming the spinal cord. The reduction of CSF space ventrally can induce strains on the spinal cord, possibly resulting in clinical affection [11]. Patient (B) shows only a right-sided anterior impression of the spinal cord, resulting in mostly unilateral or dissociated clinical symptoms [6]. Patient (C) showed a symmetrically anterior impression with deformation of the spinal cord, resulting in usually bilateral symptoms. These examples demonstrate the different impact of a primarily "relative stenosis" on the function of the spinal cord in accordance to the location of compression, resulting in heterogenic symptoms diverging from the typical dorsal cord syndrome in DCM.
