# Supplementary figures and images for: Quantification of cervical spinal stenosis by automated 3D MRI segmentation of spinal cord and cerebrospinal fluid space
Source: Spinal Cord. 2024 Apr 16;62(7):371–7. doi: 10.1038/s41393-024-00993-8 (PMC11230899; doi:10.1038/s41393-024-00993-8)

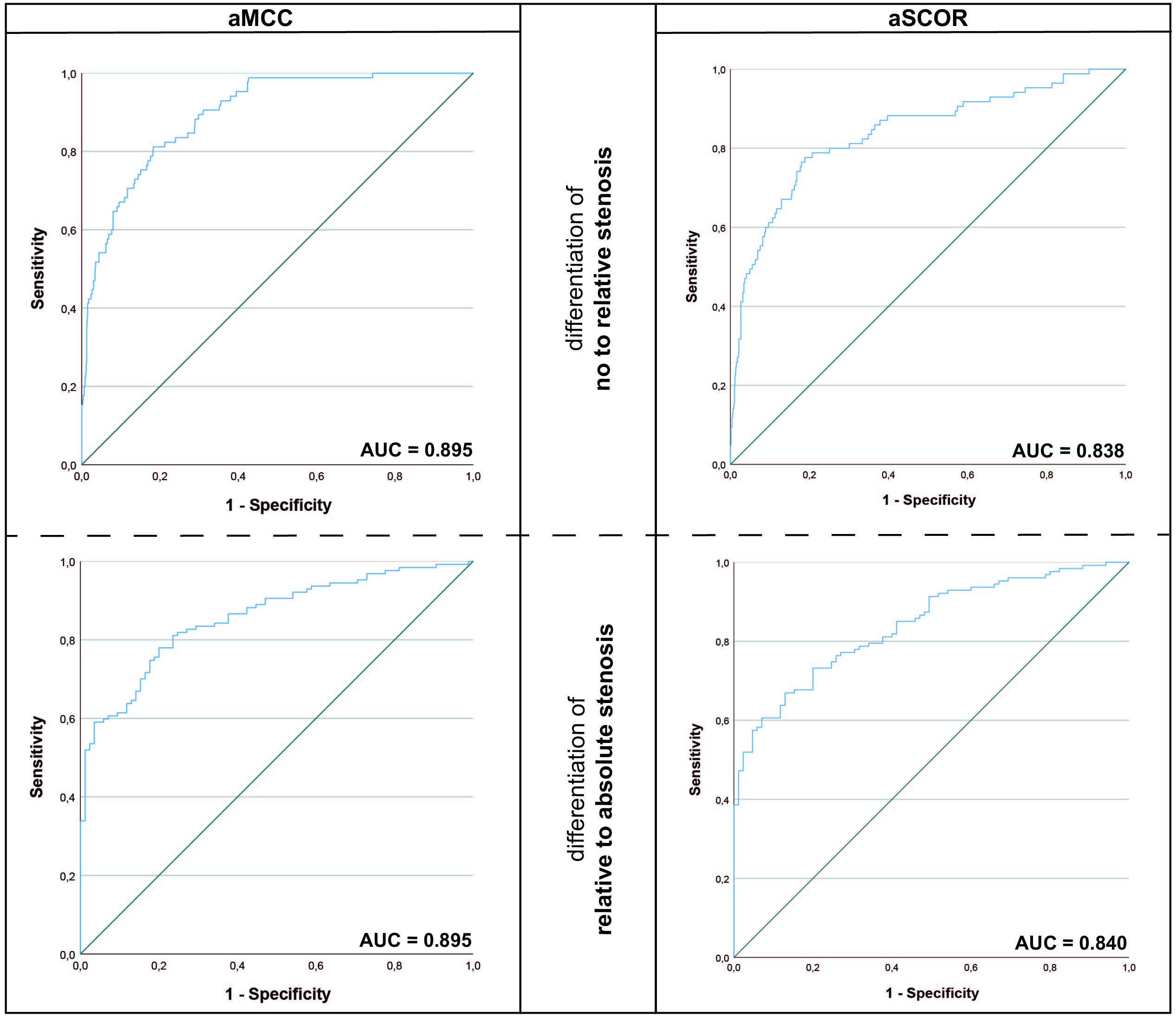

Supplement: Supplementary file 2 — Supplement 1 [file 41393_2024_993_MOESM2_ESM.jpg]

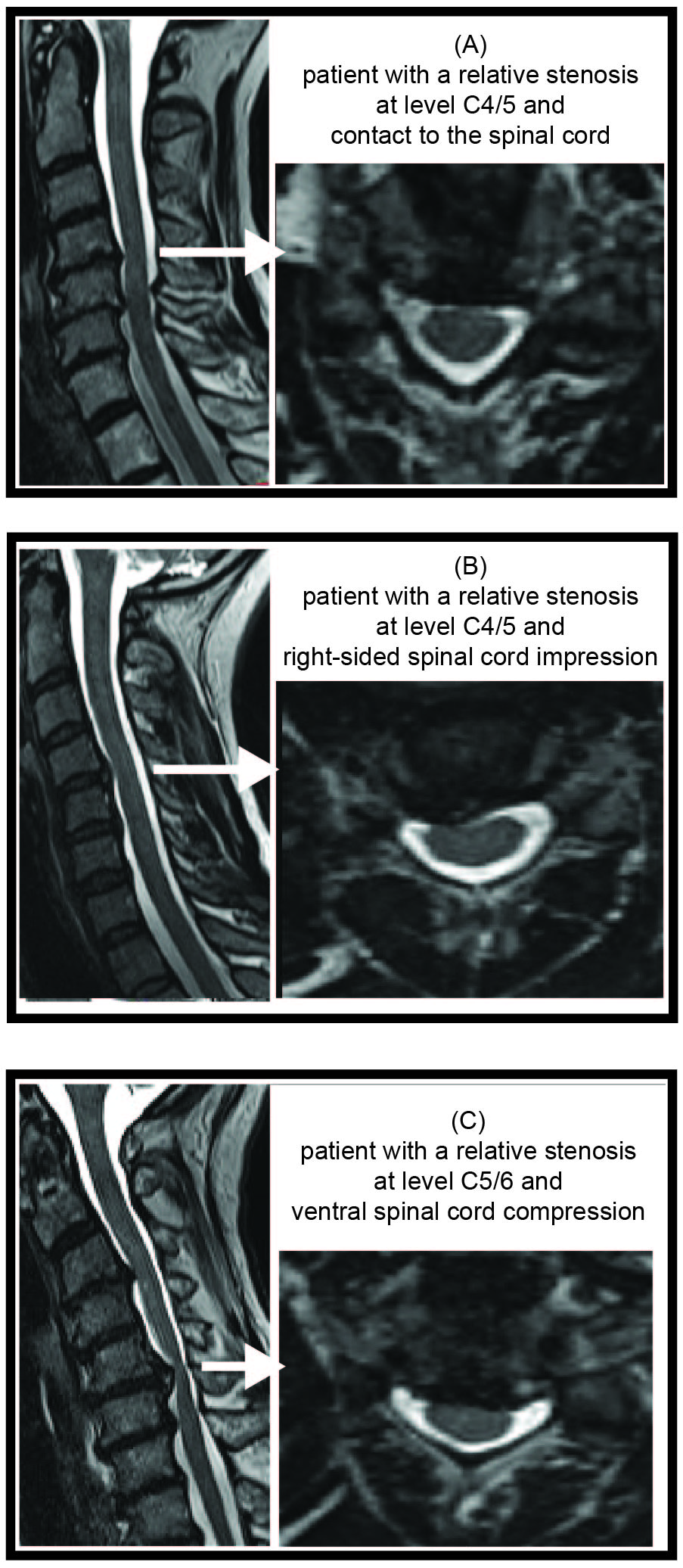

Supplement: Supplementary file 4 — Supplement 3 [file 41393_2024_993_MOESM4_ESM.jpg]
