# Supplementary material for: Quantification of cervical spinal stenosis by automated 3D MRI segmentation of spinal cord and cerebrospinal fluid space
Source: Spinal Cord. 2024 Apr 16;62(7):371–7. doi: 10.1038/s41393-024-00993-8 (PMC11230899; doi:10.1038/s41393-024-00993-8)
Supplement: Supplementary file 3 — Supplement 2 [file 41393_2024_993_MOESM3_ESM.docx]

| **Cervical Level** | **Subjective categories** | **aMCC** | | | | **aSCOR** | | | |
| --- | --- | --- | --- | --- | --- | --- | --- | --- | --- |
|  |  | **Median** | **IQR** | **p** | | **Median** | **IQR** | **p** | |
| C2/3 | No stenosis | 1.008 | 0.967 - 1.055 | --- |  | 29.049 | 25.584 - 32.585 | --- |  |
|  | Relative stenosis | --- | --- |  | --- | --- | --- |  | --- |
|  | Absolute stenosis | 1.538 | --- |  |  | 42.519 | --- |  |  |
|  |  |  |  |  |  |  |  |  |  |
| C3/4 | No stenosis | 1.080 | 1.039 - 1.151 | **<0.001** |  | 33.458 | 30.711 - 37.402 | **<0.001** |  |
|  | Relative stenosis | 1.350 | 1.268 - 1.548 |  | 0.286 | 43.187 | 35.748 - 48.512 |  | **0.026** |
|  | Absolute stenosis | 2.132 | 1.703 - 2.973 |  |  | 53.043 | 47.764 - 60.475 |  |  |
|  |  |  |  |  |  |  |  |  |  |
| C4/5 | No stenosis | 1.073 | 1.043 - 1.151 | **<0.001** |  | 33.898 | 30.878 - 38.262 | **0.011** |  |
|  | Relative stenosis | 1.237 | 1.185 - 1.273 |  | **0.046** | 39.575 | 35.010 - 51.399 |  | **0.006** |
|  | Absolute stenosis | 1.948 | 1.564 - 2.587 |  |  | 52.882 | 47.554 - 59.285 |  |  |
|  |  |  |  |  |  |  |  |  |  |
| C5/6 | No stenosis | 1.141 | 1.057 - 1.232 | **<0.001** |  | 32.884 | 30.075 - 37.241 | **<0.001** |  |
|  | Relative stenosis | 1.361 | 1.183 - 1.499 |  | **<0.001** | 43.756 | 39.897 - 47.062 |  | **0.001** |
|  | Absolute stenosis | 1.850 | 1.563 - 2.098 |  |  | 55.278 | 51.205 - 60.736 |  |  |
|  |  |  |  |  |  |  |  |  |  |
| C6/7 | No stenosis | 1.110 | 1.051 - 1.189 | **<0.001** |  | 29.938 | 26.959 - 34.041 | **<0.001** |  |
|  | Relative stenosis | 1.307 | 1.196 - 1.487 |  | 0.173 | 38.522 | 34.031 - 44.582 |  | 0.053 |
|  | Absolute stenosis | 1.817 | 1.398 - 2.260 |  |  | 48.336 | 41.875 - 54.738 |  |  |
